# Dynamic modeling of dielectric elastomer actuator with conical shape

**DOI:** 10.1371/journal.pone.0235229

**Published:** 2020-08-14

**Authors:** Peng Huang, Wenjun Ye, Yawu Wang

**Affiliations:** 1 School of Automation, China University of Geosciences, Wuhan, Hubei, China; 2 Hubei Key Laboratory of Advanced Control and Intelligent Automation for Complex Systems, Wuhan, Hubei, China; 3 Gina Cody School of Engineering and Computer Science, Concordia University, Montreal, Quebec, Canada; Tsinghua University, CHINA

## Abstract

With desirable physical performances of impressive actuation strain, high energy density, high degree of electromechanical coupling and high mechanical compliance, dielectric elastomer actuators (DEAs) are widely employed to actuate the soft robots. However, there are many challenges to establish the dynamic models for DEAs, such as their inherent nonlinearity, complex electromechanical coupling, and time-dependent viscoelastic behavior. Moreover, most previous studies concentrated on the planar DEAs, but the studies on DEAs with some other functional shapes are insufficient. In this paper, by investigating a conical DEA with the material of polydimethylsiloxane and considering the influence of inertia, we propose a dynamic model based on the principles of nonequilibrium thermodynamics. This dynamic model can describe the complex motion characteristics of the conical DEA. Based on the experimental data, the differential evolution algorithm is employed to identify the undetermined parameters of the developed dynamic model. The result of the model validation demonstrates the effectiveness of the model.

## Introduction

Soft robots, a kind of flexible machinery, aim at operating in natural environments and realizing complex functions [1]. Although conventional rigid robots have made great progress in the field of automation manufacturing, soft robots are more flexible and provide great potential applications [2]. In addition, soft robots are mostly made of soft materials, and they are capable of deforming greatly and rather adopt to the complex external environments [3, 4].

Traditional robots usually take electric motors, hydraulic motors and cylinders as their actuators. However, soft robots mostly employ the flexible actuators fabricated by soft materials [5]. The pneumatic actuator is a typical flexible actuator. In [6], a soft gripper is fabricated by full multi material 3D printing technology, which can freely deform and grip various objects. Moreover, a climbing robot designed in [7] is capable of performing 3D climbing locomotion using two suction cups. Different from the pneumatic actuator, the soft actuator based on smart materials is another typically flexible actuator. In [8], a jellyfish robot powered by the ionic polymer metal composite actuator is designed. In addition, an octopus-like robot based on the electrorheological fluid is developed in [9].

Dielectric elastomer (DE) materials are new smart materials, which have the advantages of large deformation, high energy density and fast response [10]. The dielectric elastomer actuator (DEA) is one of the most important applications of the DE material. A DEA consists of two compliant electrodes and a DE membrane that sandwiched between electrodes [11]. When a high driving voltage is applied to the electrodes, the membrane will expand in area and decrease in thickness [10, 12]. Due to the characteristic of large electrical deformation, the DEA has been employed to actuate soft robots, such as soft crawling robots [13, 14], object gripping robots [15] and fish robots [16].

The mathematical model is the basis of precisely understanding the DEA’s inherent nonlinearity, complex electromechanical coupling, and time-dependent viscoelastic behavior. Meanwhile, it is significant to explore the deformation mechanism and establish a mathematical model to describe the complex motion characteristics of the DEA. The previous studies are mostly focused on the planar DEA. In [17], a model frame is proposed to characterize the nonlinear time-dependent electromechanical response of the planar DEA.

Concerning the DEA with complex shape, some researches have been developed. In [18], a model is presented to explain the transient behavior of the cylindrically stacked DEA. In [19], a DEA subjected to the pressure and the voltage is stretched to an approximate hemispherical shape, and a mathematical model is derived to analyze its static stability and its oscillation around the state of the equilibrium. In [20], a model of the spherical DEA is built to analyze its motion characteristics when it subjects to the joint action of the pressure and the periodic voltage. In [21], a mathematical model of the spherical DEA is proposed to investigate its electromechanical instability. However, there exist DEAs with other complex shapes should be further explored.

The conical DEA is a class of DEA with complex shape. Some quasi-static models have been established to explicate the behavior of the conical DEAs. In [22], a model is proposed to characterize the quasi-static force-displacement relationship of a conical DEA. In [23], a quasi-static model is developed to predict the performance of a double cone DEA and a spring-mass model is used to characterize its natural frequency. However, the modeling processes in these papers ignore the inertial force. Meanwhile, since the DE material shows the obvious “memory” property during the dynamic response, the time-dependent viscoelastic behavior of the DEA should be fully considered. In [24, 25], the dynamic models are built to describe the complex motion characteristics of the conical DEA which is loaded by the linear spring and the biasing mass. However, the influence of inertia is neglected.

Currently, there are two common classes of DE materials: polyacrylate and polydimethylsiloxane (PDMS). 3M VHB (very high bond) tape is a kind of polyacrylate material made by 3M company, USA. The DE material employed in the previous DEA is mostly VHB, for the reason that the VHB is readily available. However, the VHB has a defect of high viscoelasticity. A promising solution of the high viscoelasticity defect is using PDMS to replace the VHB. Unfortunately, there are few researchers exploring the dynamic characteristics of the DEA based on the PDMS.

Considering both new material and complex shape, we choose the PDMS to fabricate a conical DEA. Based on the deformation mechanism of the DEA, the theory of nonequilibrium thermodynamics and the energy method, a dynamic model is developed to describe the nonlinear time-dependent viscoelastic behavior of the conical DEA. Then, an infinitesimal element with conical shape in the cylindrical coordinates is used to calculate the work done by the inertial force. To explain the stress-strain state of the DE material, Gent model is employed to describe the viscoelastic behavior of the DEA. Next, a periodic sinusoidal full wave driving voltage, whose amplitudes and frequencies can be set as different values within one period, is applied to the electrodes. Based on the experimental data, the undetermined parameters in the dynamic model are identified by the differential evolution algorithm. The model validation indicates that the dynamic model contributes to describing the viscoelastic behavior and electromechanical response of the conical DEA.

## DEA modeling

**Fig 1:**
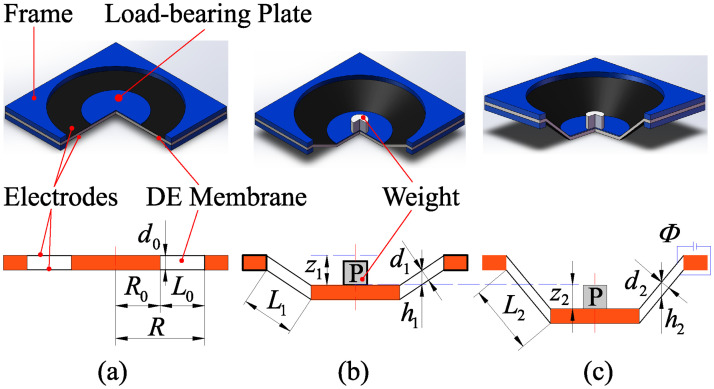
States of the DEA. (a) Un-deformed state, (b) Pre-stretched state, and (c) Electro-deformed state.

In this section, a dynamic model of a DEA with conical shape is developed. For ease of presentation, three different states of the DEA are declared in advance. The first state is called un-deformed state, the second state is called pre-stretched state, and the third state is called electro-deformed state, whose diagrams are shown in Fig 1(a), 1(b) and 1(c), respectively.

### (A) Un-deformed state

A DE membrane with thickness *d*_0_ is clamped by a frame with inner circle radius *R*. A load-bearing plate with radius *R*_0_ is placed on the center of the DE membrane. Two sides of the DE membrane, which are two annular regions, are coated with the compliant electrodes. Thus, the radial length of the DEA is *L*_0_ = *R* − *R*_0_.

### (B) Pre-stretched state

A weight with the mass *m* is placed on the center of the load-bearing plate. Subjected to the gravity *P*, the weight will move down a distance *z*_1_ to reach the equilibrium position. As a result, the DE membrane is pre-stretched as a conical shape. As shown in Fig 1(b), *L*_1_, *d*_1_ and *h*_1_ are the dimensions of the DEA corresponding to the pre-stretched state, where *L*_1_ is the generatrix length, *d*_1_ is the thickness, and *h*_1_ is the height difference between the upper surface and the lower surface.

### (C) Electro-deformed state

When a driving voltage Φ is applied to the electrodes, the DE membrane reduces in thickness and expands in area. Thus, the weight will move down a displacement *z*_2_. As shown in Fig 1(c), *L*_2_, *d*_2_ and *h*_2_ are the dimensions corresponding to the electro-deformed state.

The volumes of the DEA for the un-deformed state, the pre-stretched state and the electro-deformed state are:

$$\left\{ \begin{array}{c} V_{0}=\pi d_{0}(R^{2}-R_{0}^{2})\\ V_{1}=\pi h_{1}(R^{2}-R_{0}^{2})\\ V_{2}=\pi h_{2}(R^{2}-R_{0}^{2}) \end{array}\right.$$ (1)

Strictly speaking, the deformation of the DEA with conical shape is inhomogeneous [26, 27]. However, to simplify the dynamic modeling, the inhomogeneity of the deformation is ignored in the following development [24, 28]. Since the DEA is incompressible [29], the volume of the DEA is constant. Thus, *V*_0_ = *V*_1_ = *V*_2_. From (1), we can get

$$\begin{array}{c} d_{0}=h_{1}=h_{2} \end{array}$$ (2)

According to (2), the relationships among *z*_1_, *z*_2_, *d*_1_ and *d*_2_ are

$$\left\{ \begin{array}{c} d_{1}=h_{1}\frac{L_{0}}{L_{1}}=d_{0}\frac{L_{0}}{\sqrt{z_{1}^{2}+L_{0}^{2}}}\\ d_{2}=h_{2}\frac{L_{0}}{L_{2}}=d_{0}\frac{L_{0}}{\sqrt{{\left(z_{1}+z_{2}\right)}^{2}+L_{0}^{2}}} \end{array}\right.$$ (3)

The DEA studied in this paper is the conical shape. For ease of description, the generatrix, thickness and circumferential stretches are employed to describe the states of the DEA. In the pre-stretched state, the pre-stretches of the DEA are λ_*pre*,*L*_, λ_*pre*,*d*_ and λ_*pre*,*C*_, respectively. In the electro-deformed state, the stretches of the DEA are λ_1_, λ_2_, and λ_3_, respectively. According to Fig 1, the following equations hold:

$$\left\{ \begin{array}{c} \lambda_{pre,L}=L_{1}/L_{0}\\ \lambda_{pre,d}=d_{1}/d_{0}\\ \lambda_{pre,C}=2\pi /2\pi =1 \end{array}\right.$$ (4)

{λ1=L2/L2L0L0λ2=d2/d2d0d0λ3=2π/2π2π=2π=1 (5)

According to (2)–(5), the following equation is established:

$$\begin{array}{c} \lambda_{1}\lambda_{2}\lambda_{3}=\lambda_{pre,L}\lambda_{pre,d}\lambda_{pre,C}=1 \end{array}$$ (6)

The relationship between the charge *Q* and the voltage Φ is

$$\begin{array}{c} Q=\Phi C=\Phi \frac{\varepsilon \pi L_{2}(R+R_{0})}{d_{2}}=\frac{\varepsilon \Phi \pi (R^{2}-R_{0}^{2})\lambda_{1}^{2}}{d_{0}} \end{array}$$ (7)

where *ε* and *C* are the permittivity and the capacitance of the DE material, respectively.

According to (3)–(6), the relationship between *δλ*_1_ and *δz*_2_ is

$$\begin{array}{c} \frac{\delta z_{2}}{\delta \lambda_{1}}=\frac{L_{2}L_{0}}{\sqrt{L_{2}^{2}-L_{0}^{2}}} \end{array}$$ (8)

From (6) and (7), the charge on the electrode varies by

$$\begin{array}{c} \delta Q=\frac{\varepsilon \pi (R^{2}-R_{0}^{2})}{d_{0}}(\lambda_{1}^{2}\delta \Phi +2\Phi \delta \lambda_{1}) \end{array}$$ (9)

**Fig 2:**
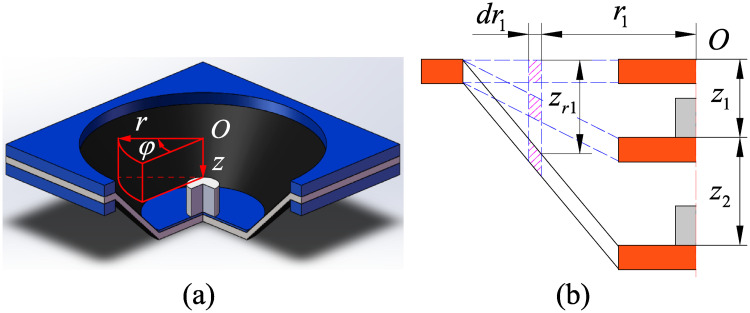
Displacement of element in cylindrical coordinates: (a) Cylindrical coordinates, and (b) Displacement of element in each state.

To calculate the work of the inertial forces during the electromechanical deformation, we consider the cylindrical coordinates shown in Fig 2, where *O*, *r*, *φ* and *z* represent the coordinate origin, the radial distance, the azimuth angle and the height of the cylindrical coordinates, respectively.

As shown in Fig 2(b), an infinitesimal element with inner radius *r*_1_ and outside radius *r*_1_+ *dr*_1_ is investigated. In the electro-deformed state, the displacement of the element along the *r*-direction, *φ*-direction, and *z*-direction are 0, 0 and *z*_*r*1_, respectively. So, the relationship between *z*_*r*1_ and *z*_2_ is

$$\begin{array}{c} z_{r1}=(z_{1}+z_{2})\frac{R-r_{1}}{R-R_{0}} \end{array}$$ (10)

The inertial forces in each material element along the *r*-direction, *φ*-direction, and *z*-direction are 0, 0 and *dF*_*r*1_, respectively. According to D’Alembert’s principle, we can get

$$\begin{array}{c} dF_{r1}=-\rho \cdot 2\pi d_{0}r_{1}dr_{1}\cdot \frac{d^{2}z_{r1}}{dt^{2}} \end{array}$$ (11)

where *ρ* is the density of the DE material.

Thus, the changes of works done by the inertial forces are 0, 0 and *δH*_*I*,*z*_, respectively. According to (10) and (11), the work done by the inertial force *dF*_*r*1_ is

$$\begin{array}{c} \delta H_{I,z}=\int_{R_{0}}^{R}\delta z_{r1}dF_{r1}=-\frac{\rho \pi d_{0}L_{0}(R+3R_{0})}{6}\frac{d^{2}z_{2}}{dt^{2}}\delta z_{2} \end{array}$$ (12)

The change of the free energy of the DEA is equal to the sum of the works done by the driving voltage, the gravity and the inertial forces. That is,

$$\begin{array}{c} \pi d_{0}(R^{2}-R_{0}^{2})\delta W=\Phi \delta Q+P\delta z_{2}+(0+0+\delta H_{I,z}) \end{array}$$ (13)

where *W* is the free energy density of the DEA, and *δW* represents the change of *W*.

By submitting (9) and (12) into (13), the free energy density *W* varies by

$$\begin{array}{c} \delta W=\frac{\varepsilon \Phi (\lambda_{1}^{2}\delta \Phi +2\Phi \lambda_{1}\delta \lambda_{1})}{d_{0}^{2}}+\frac{P\delta z_{2}}{\pi d_{0}(R^{2}-R_{0}^{2})}\\ \;\;\;\;\;\;\;\;\;\;\;-\frac{\rho (R+3R_{0})}{6(R+R_{0})}\frac{d^{2}z_{2}}{dt^{2}}\delta z_{2} \end{array}$$ (14)

Submitting (8) into (14), we can get

$$\begin{array}{c} \frac{\partial W}{\partial \lambda_{1}}=\frac{2\varepsilon \Phi^{2}\lambda_{1}}{d_{0}^{2}}+\frac{PL_{2}}{\pi d_{0}(R+R_{0})\sqrt{L_{2}^{2}-L_{0}^{2}}}\\ \;\;\;\;\;\;\;\;\;\;\;\;-\frac{\rho L_{2}L_{0}(R+3R_{0})}{6(R+R_{0})\sqrt{L_{2}^{2}-L_{0}^{2}}}\frac{d^{2}z_{2}}{dt^{2}} \end{array}$$ (15)

where

$$\begin{array}{c} \frac{d^{2}z_{2}}{dt^{2}}=\frac{-L_{0}^{4}}{{\left(L_{2}^{2}-L_{0}^{2}\right)}^{\frac{3}{2}}}{\left(\frac{d\lambda_{1}}{dt}\right)}^{2}+\frac{L_{2}L_{0}}{\sqrt{L_{2}^{2}-L_{0}^{2}}}\frac{d^{2}\lambda_{1}}{dt^{2}} \end{array}$$ (16)

**Fig 3:**
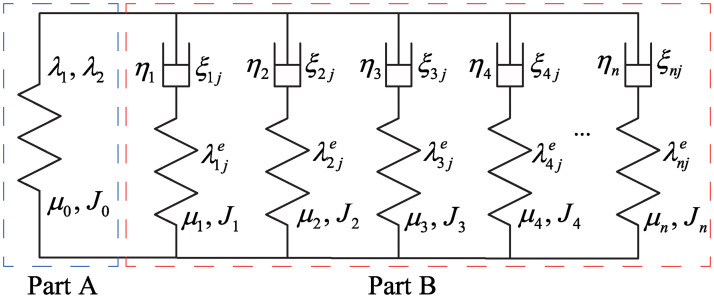
Rheological model: Part A only consists of a spring, but each unit of Part B consists of a spring with a series-wound dashpot.

In order to describe the viscoelasticity of the DE material, the rheological model with two parallel units (as shown in Fig 3) is adopted [30]. The part A only consists of a spring *α*_0_, while the part B consists of four parallel formations and each formation consists of a spring *α_i_*(*i* = 1, 2, 3, 4, …, *n*) with a series-wound dashpot. In this paper, we suppose each dashpot to be a Newtonian fluid with viscosity *η*_*i*_. Let *ξ_ij_* (*j* = 1, 2) be the stretches due to the dashpots, the stretches of the spring *α*_*i*_ are determined by multiplication rules $\lambda_{i1}^{e}=\lambda_{1}/\xi_{i1}$, and $\lambda_{i2}^{e}=\lambda_{2}/\xi_{i2}=\lambda_{1}^{-1}\xi_{i2}^{-1}$.

The free energy density *W* of the DEA [29] can be described as

$$\begin{array}{c} W=W_{s}+\frac{D^{2}}{2\varepsilon } \end{array}$$ (17)

where *W*_*s*_ is the Helmholtz free energy associated with the stretching of the elastomer, and *D* is the electric displacement. The electric displacement *D* is equal to

$$\begin{array}{c} D=\frac{Q}{\pi L_{2}(R+R_{0})} \end{array}$$ (18)

In this paper, we choose Gent model [17, 30] to describe the elastic energy density of the DEA. Therefore, the elastic energy density of the DEA is

$$\begin{array}{c} W_{\text{s}}=\sum_{i=0}^{n}W_{ela}^{\alpha_{i}}=-\frac{\mu_{0}J_{0}}{2}\ln(1-\frac{\lambda_{1}^{2}+\lambda_{2}^{2}+\lambda_{1}^{-2}\lambda_{2}^{-2}-3}{J_{0}})\\ \;\;\;\;\;\;\;\;\;\;\;\;\;\;\;\;-\sum_{i=1}^{n}\frac{\mu_{i}J_{i}}{2}\ln(1-\frac{\lambda_{1}^{2}\xi_{i1}^{-2}+\lambda_{2}^{2}\xi_{i2}^{-2}+\lambda_{1}^{-2}\lambda_{2}^{-2}\xi_{i1}^{2}\xi_{i2}^{2}-3}{J_{i}}) \end{array}$$ (19)

where $W_{ela}^{\alpha_{i}}$ are elastic energy densities of the spring *α*_*i*_; *μ*_*i*_ are shear modulus of the spring *α*_*i*_, respectively; *J*_*i*_ are deformation limits of the spring *α*_*i*_, respectively.

According to (5)–(7) and (17)–(19), the free energy density of the DEA is

$$\begin{array}{c} W=\frac{\varepsilon \Phi^{2}\lambda_{1}^{2}}{2d_{0}^{2}}-\frac{\mu_{0}J_{0}}{2}\ln(1-\frac{\lambda_{1}^{2}+\lambda_{1}^{-2}-2}{J_{0}})\\ \;\;\;\;\;\;\;\;\;\;\;\;\;\;\;\;-\sum_{i=1}^{n}\frac{\mu_{i}J_{i}}{2}\ln(1-\frac{\lambda_{1}^{2}\xi_{i1}^{-2}+\lambda_{1}^{-2}\xi_{i2}^{-2}+\xi_{i1}^{2}\xi_{i2}^{2}-3}{J_{i}}) \end{array}$$ (20)

According to Newton’s third law of motion, the stresses of the spring *α_i_*(*i* = 1, 2, 3, 4, …, *n*) are equal to the corresponding stresses of the dashpot. So,

$$\begin{array}{c} -\xi_{ij}\frac{\partial W_{ela}}{\partial \xi_{ij}}=\eta_{i}\frac{d\xi_{ij}}{dt}\;\;\;\;\;\;\;(i=1,\;2,\;\ldots ,\;n;\;j=1,\;\;2) \end{array}$$ (21)

From (19) and (21), the strain rates of the dashpots can be expressed as

$$\left\{ \begin{array}{c} \frac{d\xi_{i1}}{dt}=-\frac{\mu_{i}}{\eta_{i}}\frac{-\lambda_{1}^{2}\xi_{i1}^{-2}+\xi_{i1}^{2}\xi_{i2}^{2}}{1-\frac{\lambda_{1}^{2}\xi_{i1}^{-2}+\lambda_{1}^{-2}\xi_{i2}^{-2}+\xi_{i1}^{2}\xi_{i2}^{2}-3}{J_{i}}}\\ \frac{d\xi_{i2}}{dt}=-\frac{\mu_{i}}{\eta_{i}}\frac{-\lambda_{1}^{-2}\xi_{i2}^{-2}+\xi_{i1}^{2}\xi_{i2}^{2}}{1-\frac{\lambda_{1}^{2}\xi_{i1}^{-2}+\lambda_{1}^{-2}\xi_{i2}^{-2}+\xi_{i1}^{2}\xi_{i2}^{2}-3}{J_{i}}} \end{array}\right.$$ (22)

The viscoelastic relaxation time *T*_*i*_ (*i* = 1, 2, …, *n*) of the DEA is defined as the ratio of *η*_*i*_ to *μ*_*i*_. So,

Ti(t)=ηi/ηiμiμi (23)

Submitting (20) into (15), and combining the result with (22), the dynamic model of the conical DEA can be described as

$$\left\{ \begin{array}{c} \frac{\rho \lambda_{1}^{2}L_{0}^{2}(R+3R_{0})}{6(R+R_{0})(\lambda_{1}^{2}-1)}\frac{d^{2}\lambda_{1}}{dt^{2}}=\frac{\rho \lambda_{1}L_{0}^{2}(R+3R_{0})}{6(R+R_{0}){\left(\lambda_{1}^{2}-1\right)}^{2}}{\left(\frac{d\lambda_{1}}{dt}\right)}^{2}+\frac{P\lambda_{1}}{\pi d_{0}(R+R_{0})\sqrt{\lambda_{1}^{2}-1}}\\ \;+\frac{\varepsilon \Phi^{2}\lambda_{1}}{d_{0}^{2}}-\mu_{0}\frac{\lambda_{1}-\lambda_{1}^{-3}}{1-\frac{\lambda_{1}^{2}+\lambda_{1}^{-2}-2}{J_{0}}}-\sum_{i=1}^{n}\mu_{i}\frac{\lambda_{1}\xi_{i1}^{-2}-\lambda_{1}^{-3}\xi_{i2}^{-2}}{1-\frac{\lambda_{1}^{2}\xi_{i1}^{-2}+\lambda_{1}^{-2}\xi_{i2}^{-2}+\xi_{i1}^{2}\xi_{i2}^{2}-3}{J_{i}}}\\ \frac{d\xi_{i1}}{dt}=-\frac{\mu_{i}}{\eta_{i}}\frac{-\lambda_{1}^{2}\xi_{i1}^{-2}+\xi_{i1}^{2}\xi_{i2}^{2}}{1-\frac{\lambda_{1}^{2}\xi_{i1}^{-2}+\lambda_{1}^{-2}\xi_{i2}^{-2}+\xi_{i1}^{2}\xi_{i2}^{2}-3}{J_{i}}}\\ \frac{d\xi_{i2}}{dt}=-\frac{\mu_{i}}{\eta_{i}}\frac{-\lambda_{1}^{-2}\xi_{i2}^{-2}+\xi_{i1}^{2}\xi_{i2}^{2}}{1-\frac{\lambda_{1}^{2}\xi_{i1}^{-2}+\lambda_{1}^{-2}\xi_{i2}^{-2}+\xi_{i1}^{2}\xi_{i2}^{2}-3}{J_{i}}}\;(i=1,2,3,4,\cdots ,n) \end{array}\right.$$ (24)

So far, we have developed a dynamic model to describe the inherent nonlinearity, complex electromechanical coupling and time-dependent viscoelastic behavior of the conical DEA. In the following works, we conduct experiment to acquire experimental data of the conical DEA, and then employ differential evolution algorithm to identify the undetermined parameters in (24) based on these data.

## System description

In this section, we first introduce the fabrication of the conical DEA briefly. Then, the experimental platform is described.

### DEA fabrication

**Fig 4:**
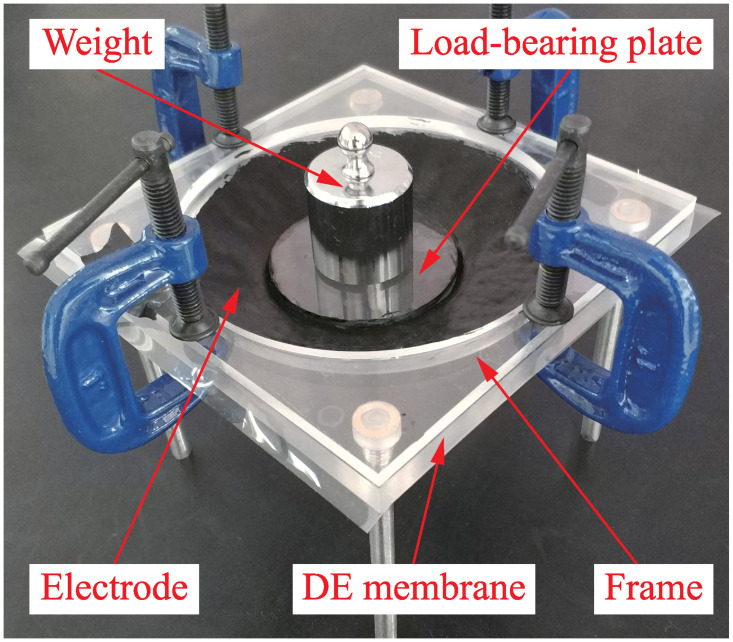
Structure of conical DEA, which mainly includes DE membrane, frame, load-bearing plate, electrode and weight.

A conical DEA is fabricated as shown in Fig 4. It’s mainly assembled by five components: (1) DE membrane (Material: PDMS; Manufacturer: Wacker Chemie AG, Germany; Undeformed thickness: *d*_0_ = 200*μ*m). (2) Frame (Material: Polymethyl methacrylate (PMMA); Inner circle radius: *R* = 6cm). (3) Load-bearing plate (Material: PMMA; Radius: *R*_0_ = 3cm). (4) Electrode (Material number: DD-10; Manufacturer: Saidi Technology, China). (5) Weight (Mass: *m* = 200g).

It is worth pointing out that the DE film has been wrinkled before applying voltage, and such wrinkles are difficult to be eliminated completely. However, we repeatedly adjusted the pose of the load-bearing plate to eliminate the wrinkles of the DE film as much as possible. Meanwhile, the DEA was left standing for a long time to make the wrinkles eliminate gradually before each experiment. Through the above measures, the DE film has fewer wrinkles in the experiment, the effect of wrinkling on the electrical deformation is minimized, and the availabilities of experimental results are ensured.

### Experimental platform

**Fig 5:**
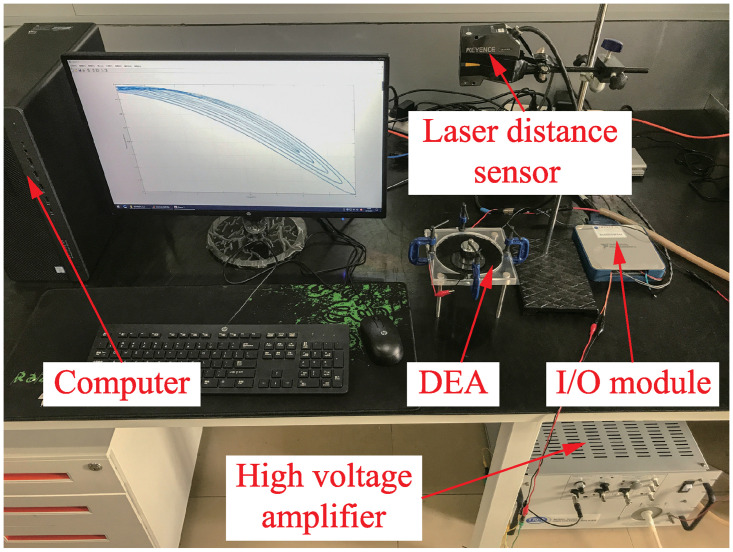
Picture of experimental platform, which mainly includes computer, high voltage amplifier, laser distance sensor, I/O module and conical DEA.

The experimental platform (see Fig 5) consists of four components: (1) High voltage amplifier (Model number: 10/40A-HS-H-CE; Manufacturer: TREK, USA); (2) Laser distance sensor (Model number: LK-H152; Manufacturer: Keyence, Japan); (3) I/O module (Model number: PCIe-6361; Manufacturer: National Instruments, USA). (4) Computer (CPU: i7-8700; Memory: 16G; Manufacturer: Hewlett Packard, USA).

The function of the I/O module is to output an original voltage signal for the high voltage amplifier, and capture the real-time displacement data from the laser sensor. The high voltage amplifier is used to amplify the original voltage signal by 1000 times and apply it to the electrodes of the DEA.

## Model identification

In this section, we first introduce the driving voltage applied in the experiment. Then, the undetermined parameters are identified based on the differential evolution algorithm. Considering the precision and the hardware capabilities, in the dynamic model (24), four spring-dashpot units are employed to describe the viscoelasticity of the DEA.

### Driving voltage

To facilitate the acquisitions of the experimental data, the following driving voltage is applied.

{tm=rem(t,∑i=151/fi)v(tm)=a1sin(f1πtm),0⩽tm⩽1/f1v(tm)=a2sin(f2πtm-f2π/f2πf1f1),1/f1⩽tm⩽∑i=121/fiv(tm)=a3sin(f3πtm-f3π∑i=121/fi),∑i=121/fi⩽tm⩽∑i=131/fiv(tm)=a4sin(f4πtm-f4π∑i=131/fi),∑i=131/fi⩽tm⩽∑i=141/fiv(tm)=a5sin(f5πtm-f5π∑i=141/fi),∑i=141/fi⩽tm⩽∑i=151/fi (25)

where *a*_*i*_ is amplitude; *f*_*i*_ is the frequency; *t* is the time; *rem*(*α*/*β*) is the remainder of *α* divided by *β*. By letting $t_{m}=rem(t,\sum_{i=1}^{5}1/f_{i})$, the periodic driving voltage in *t* ∈ [0, + ∞) is generated. By setting different values of *a*_*i*_ and *f*_*i*_, the driving voltages with different amplitudes and different frequencies are generated within one period.

### Parameters identification

**Fig 6:**
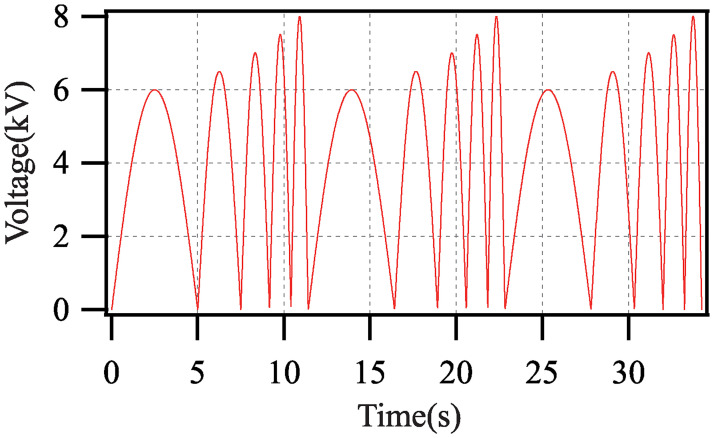
Diagram of driving voltage applied in parameters identification.

In the pre-stretched state, the vertial displacement of the weight is measured to be *z*_1_ = 1.26 (cm). The sampling period of the experiment is set as *T* = 0.01 (s). When *a*_*i*_ = 5.5+ 0.5*i* (kV) (*i* = 1, 2, …, 5) and *f*_*i*_ = 0.2*i* (Hz), the diagram of the driving voltage is shown in Fig 6. To avoid the negative displacement of the weight, the maximum frequency of the driving voltage is limited to 1.0 (Hz) in all experiments [31].

**Fig 7:**
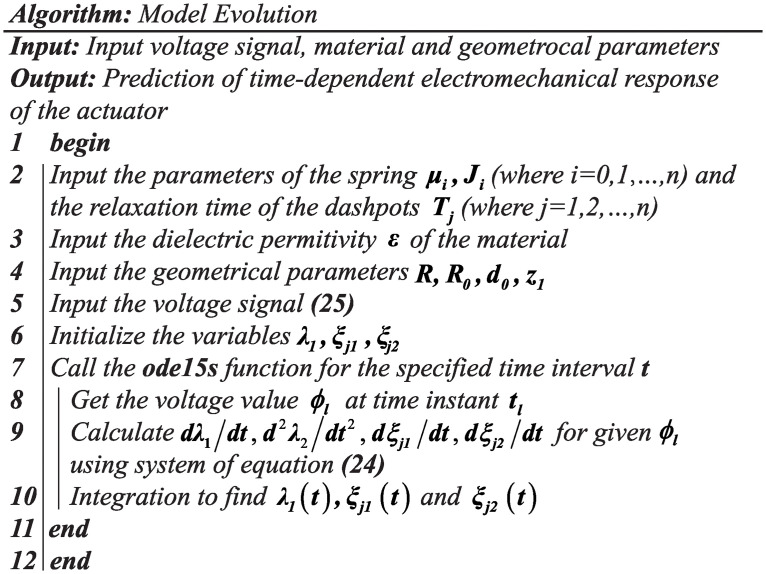
Algorithm.

The differential evolution algorithm for the parameters identification is briefly listed in the Fig 7. Considering that we do not have any prior knowledge about the values of *J*_*i*_, *μ*_*i*_ and *T*_*i*_, we set the large enough search ranges to ensure that the differential evolution algorithm could find out the optimal solution. That is, the search range of *J*_*i*_ is (0, 9 × 10^8^], the search range of *μ*_*i*_ is (0, 8 × 10^6^] and the search range of *T*_*i*_ is (0, 3 × 10^6^].

For conveniently describing the performance of the model prediction, the root-mean-square error *e*_*rms*_ and the maximum tracking error *e*_*m*_ are introduced.

$$\left\{ \begin{array}{c} e_{rms}=\sqrt{\frac{1}{n}\sum_{i}^{n}{\left(z_{ei}-z_{mi}\right)}^{2}}\times 100\%\\ e_{m}=\frac{\max(|z_{ei}-z_{mi}|)}{\max(z_{mi})-\min(z_{mi})}\times 100\% \end{array}\right.$$ (26)

where *z*_*ei*_ and *z*_*mi*_ represent the experimental data and the model predicted value of the displacement in the vertical direction; *n* is the sampling quantity within the sampling time.

**Fig 8:**
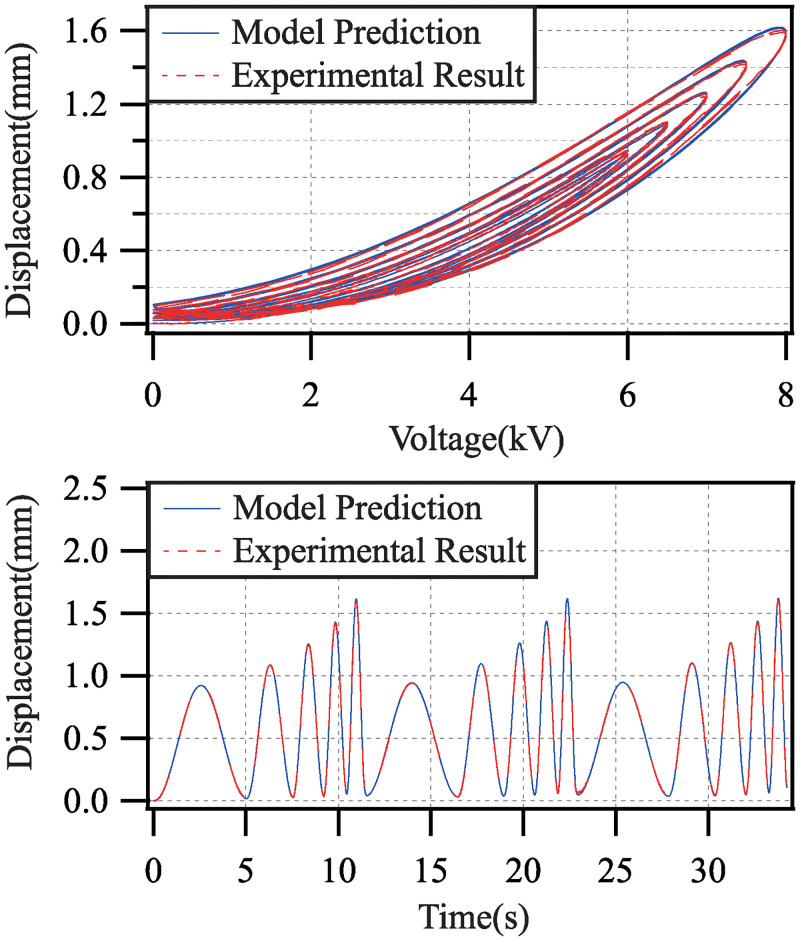
Comparison of model prediction and experimental result with different driving voltage amplitudes and different frequencies.

**Fig 9:**
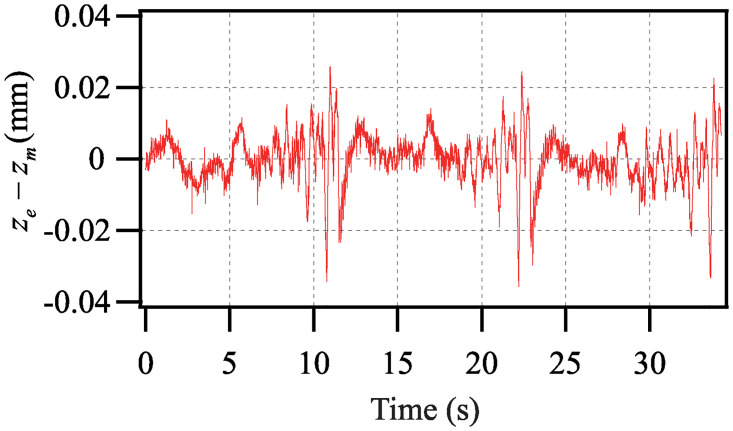
Error between model prediction and experimental result.

Fig 8 shows the comparison of the model prediction and the experimental result. The error between the model prediction and the experimental result *z*_*e*_ − *z*_*m*_ is shown in Fig 9. Table 1 lists the identified parameters of the dynamic model (24). The root-mean-square error *e*_*rms*_ is 0.69% and the maximum tracking error *e*_*m*_ is 1.60%.

**Table 1:** Parameters of the DEA model.

*i*	*μ*_*i*_(kPa)	*J*_*i*_	*T_i_* = *η_i_*/*μ_i_*
*i* = 0	0.1	6.8 × 10^7^	—
*i* = 1	5277.4	79.9 × 10^7^	0.01
*i* = 2	0.1	80.1 × 10^7^	3945.71
*i* = 3	33.1	7.9 × 10^7^	9.82
*i* = 4	571.2	3.7 × 10^7^	8484.73

## Model validation

The input of the dynamic model (24) is the voltage shown in (25). By setting different values of *a*_*i*_ and *f*_*i*_, in this section, the generalization ability of the proposed dynamic model of the conical DEA is validated.

### Model validation with different driving voltage amplitudes

The amplitudes of the driving voltage are set to be *a*_*i*_ = 5.5+ 0.5*i* (kV) (*i* = 1, 2, …, 5). Moreover, the frequencies are set to be *f*_*i*_ = 0.2, 0.4, 0.6, 0.8, 1.0 (Hz), respectively. So, the driving voltage has various amplitudes but single frequency in each test experiment.

**Fig 10:**
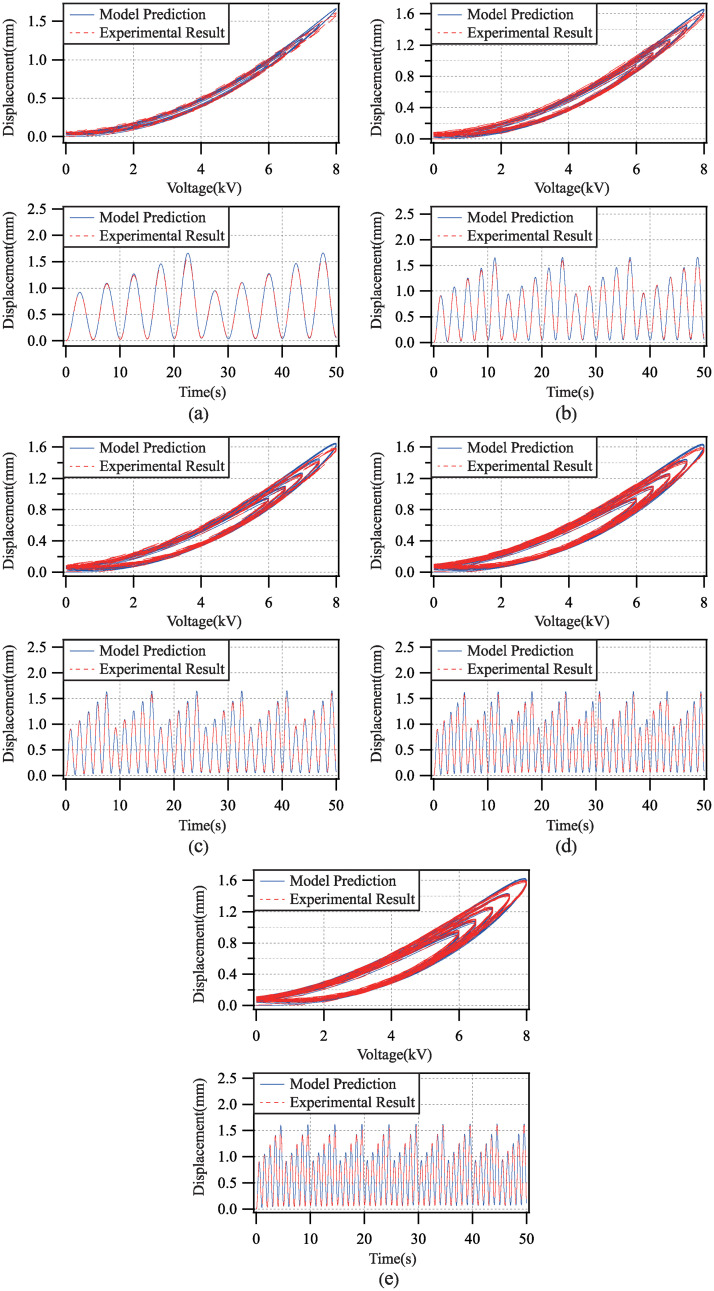
Comparisons of model prediction and experimental result with driving voltage frequency 0.2(Hz), 0.4(Hz), 0.6(Hz), 0.8(Hz) and 1.0(Hz).

Applied by the driving voltage with single frequency and multi amplitudes, the comparisons of the model prediction and the experimental result in each test experiment are shown in Fig 10. The modeling error for all test experiments are shown in Table 2.

**Table 2:** Errors of model validation with different driving voltage amplitudes.

*i*	*e*_*rms*_	*e*_*m*_
*f* = 0.2	1.7734	5.4161
*f* = 0.4	1.6875	4.1992
*f* = 0.6	1.7013	4.3098
*f* = 0.8	1.3202	3.6769
*f* = 1.0	1.2557	2.6517

According to the above results, the root-mean-square error of the modeling for any test experiment is less than 3%, and the maximum modeling error for any test experiment is less than 6%. Therefore, the generalization ability of the proposed dynamic model of the DEA is fairly good.

### Model validation with different driving voltage frequencies

The amplitudes of the driving voltage are set to *a*_*i*_ = 6.0, 6.5, 7.0, 7.5, 8.0 (kV), respectively. Meanwhile, the frequencies are set to be *f*_*i*_ = 0.2*i* (Hz) (*i* = 1, 2, …, 5). Thus, the driving voltage has various frequencies but single amplitude in each test experiment.

**Fig 11:**
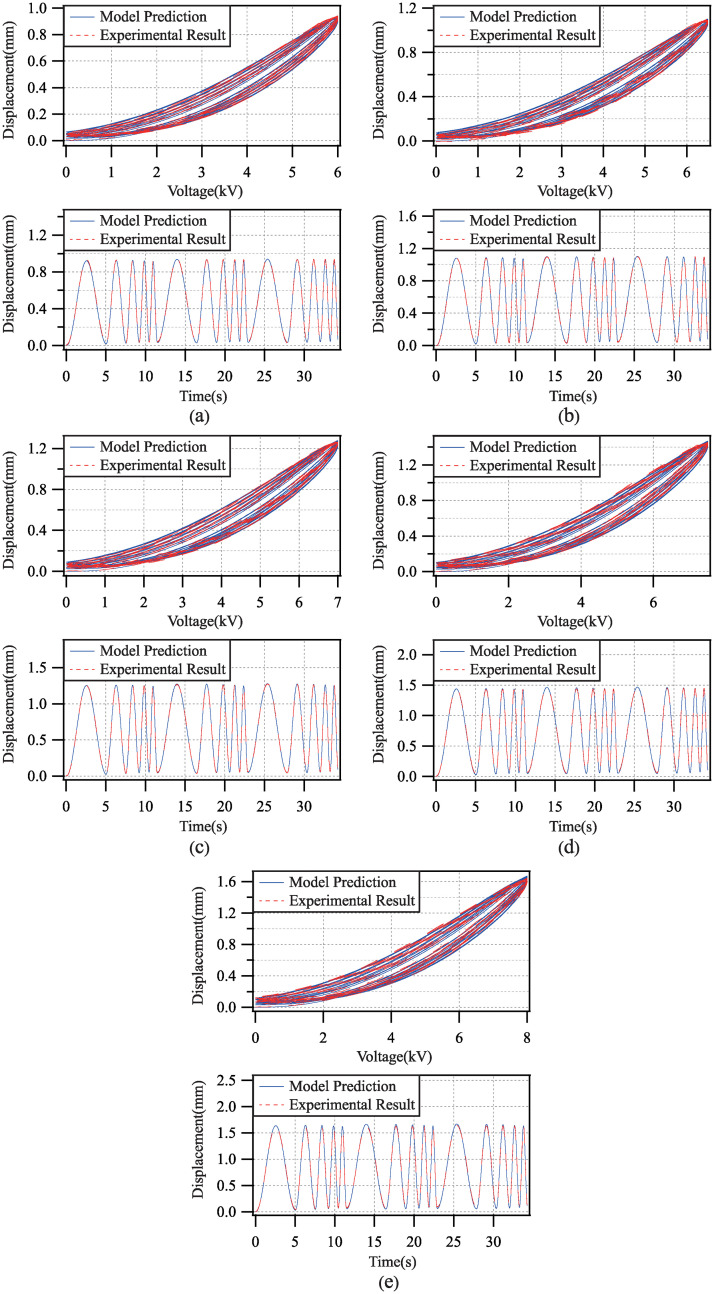
Comparisons of model prediction and experimental result with driving voltage amplitude 6.0(kV), 6.5(kV), 7.0(kV), 7.5(kV) and 8.0(kV).

Applied by the driving voltage with single amplitude and multi frequencies, the comparisons of the model prediction and the experimental result in each test experiment are shown in Fig 11. The modeling error for all test experiments are shown in Table 3.

**Table 3:** Errors of model validation with different driving voltage frequencies.

*i*	*e*_*rms*_	*e*_*m*_
*a* = 6.0	0.6047	1.7454
*a* = 6.5	0.7842	2.2600
*a* = 7.0	0.6878	1.5985
*a* = 7.5	1.2279	1.6069
*a* = 8.0	1.8889	2.5015

According to the above results, the root-mean-square error of the modeling for any test experiment is less than 2%, and the maximum modeling error for any test experiment is less than 3%. Therefore, the developed dynamic model has excellent performance in the generalization ability.

### Model validation corresponding to force analysis

To further verify the validation of the proposed model, the force versus displacement and force versus voltage tests for the dynamic response are performed. The amplitudes and the frequencies of the driving voltage are chosen to be *a*_*i*_ = 5.5+ 0.5*i*(*i* = 1, 2, …, 5) (kV) and *f*_*i*_ = 0.2*i* (Hz), respectively. Based on the real-time displacement data measured by the laser sensor, the accelerated velocity of the weight is calculated by adopting the third-order differentiator. Thus, the output force of the DEA for the experiment can be obtained according to Newton’s second law. Moreover, the output force with respect to the model prediction can be calculated according to the proposed model (24).

**Fig 12:**
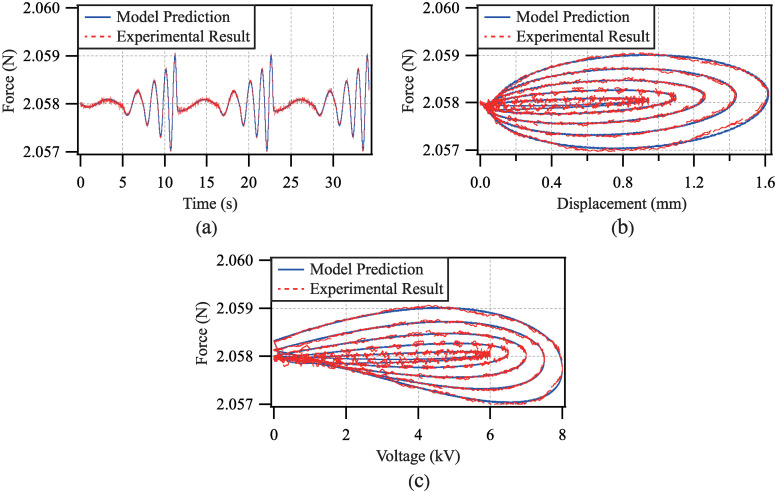
Comparisons of model prediction and experimental result corresponding to: (a) force versus time, (b) force versus displacement, and (c) force versus voltage.

In this way, the comparisons of the model prediction and the experimental result corresponding to the force versus time, force versus displacement and force versus voltage are given in Fig 12. The root-mean-square error and the maximum tracking error for all tests are 0.0028% and 6.0011%, respectively. Therefore, the validation of the proposed dynamic model is further verified.

In the above works, we verify the validity of the model driving by the voltage with different amplitudes and frequencies, respectively. Meanwhile, the force versus displacement and force versus voltage analyses are conducted. According to the comparison results, the proposed dynamic model is valid.

**Fig 13:**
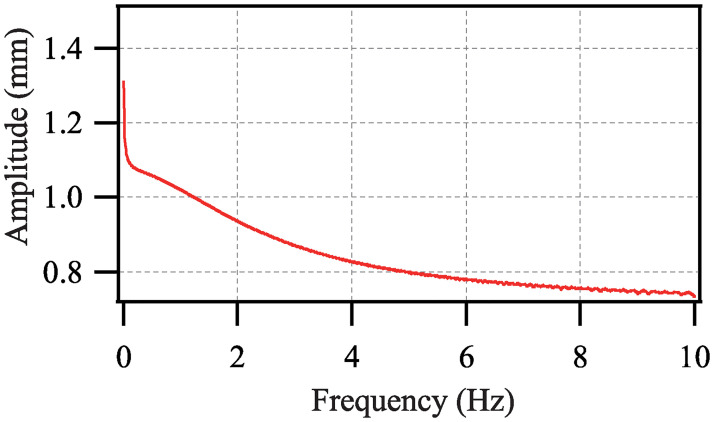
Amplitude-frequency response curve.

Next, to further reflect the value of the model, the amplitude-frequency response analysis is developed. The sinusoidal voltages with frequencies 0.01 Hz to 10 Hz (spacing 0.01 Hz) are employed in the theoretical calculations. The amplitude-frequency response curve is shown in Fig 13. With the increase of the frequency of the driving voltage, the amplitude of the conical DEA reduces continuously. This may originate from the viscoelasticity of the DE material [23].

## Conclusion

In this paper, the dynamic model of the conical DEA is proposed based on the theory of nonequilibrium thermodynamics. First, three different states of the DEA are declared and its deformation mechanism is analyzed. Then, the infinitesimal element with conical shape in cylindrical coordinates is used to calculate the work done by the inertial force. To describe the elastic energy and the viscoelasticity of the DEA, Gent model and the rheological model are employed respectively. Next, the undetermined parameters in the dynamic model of the DEA are identified by using the differential evolution algorithm. Finally, the comparisons of the experimental result and the model prediction output demonstrate that the proposed dynamic model can describe the inherent nonlinearity, complex electromechanical coupling and time-dependent viscoelastic behavior of the conical DEA. In addition, we find that the DEA shows the obvious hysteresis behavior, creep behavior, and even rate-dependence hysteresis behavior during the experiments. The proposed model can still handle the above behaviors accurately. So, the dynamic model contributes to understanding the complex motion characteristics of the conical DEA.
